# Thyroid cancer causing obstruction of the great veins in the neck

**DOI:** 10.1186/1477-7819-6-36

**Published:** 2008-04-03

**Authors:** Steve L Hyer, Prasad Dandekar, Kate Newbold, Masud Haq, Kshama Wechalakar, Clive Harmer

**Affiliations:** 1 Thyroid Unit, Royal Marsden Hospital, Fulham Road, London, SW3 6JJ, UK

## Abstract

**Background and aims:**

To report our experience and review the literature of thyroid cancer obstructing the great veins in the neck, highlighting clinical aspects and response to treatment.

**Methods:**

Clinical data were collected from the thyroid cancer register and from follow-up clinic visits of patients referred to the Thyroid Unit at the Royal Marsden Hospital. A Medline literature search was conducted between 1980 and 2007.

**Results:**

Of 1448 patients with thyroid cancer on our cancer register and treated in our unit over the last 60 years, we identified five patients, four women and one man, aged 43 – 81 years with a median follow up of 28 (24–78) months in whom tumour had occluded the great veins in the neck. All patients underwent total thyroidectomy and all subsequently received ablative ^131^I with the exception of patient 3 whose post-operative isotope scan shown no significant ^131^I uptake. External beam radiotherapy to the neck and upper mediastinum was used for residual disease control in the 5 patients. The median survival was 28 months and the disease-free survival was 24 months. One patient remains asymptomatic but with disease 53 months after initial presentation. Survival in this small series is significantly better than that previously reported for this condition.

**Conclusion:**

A multimodality therapeutic approach comprising surgery, radioiodine and external beam radiotherapy may give the best results for patients in whom thyroid cancer is occluding the great veins.

## Background

Microscopic vascular invasion is well recognized in thyroid cancer particularly in the follicular and poorly differentiated histological types [1]. However massive invasion of tumour into the great veins or external compression of the superior vena cava is rare. Only 24 cases have been documented in the literature (Table 1). Management of these patients is challenging as they typically present with advanced and rapidly progressive disease [2,3]. We identified five patients from our thyroid cancer register with occlusion of the great veins by tumour who were managed at our centre. Clinical features, management and outcome of intervention are presented here together with a review of the literature.

**Table 1:** Reported cases of invasion or occlusion of great veins by thyroid cancer since 1930

Study	Gender	Age	Signs SVCO/dilated veins	Diagnosis	Pathology	Extension	Treatment	Outcome
Wylegschanin (1930) [17]	F	52	Yes	At autopsy	Follicular cell carcinoma	JV, BV, SVC, RA		Died 2 months
Holt (1934) [5]	M	72	Yes	At autopsy	Adeno-carcinoma	JV, BV, SCV		Died 5 days
Mencarelli (1934) [17]	M	56	Yes	At autopsy	Anaplastic carcinoma	JV, RV		Sudden death
Kim (1966) [6]	M	64	Yes	At autopsy	Follicular cell carcinoma	JV, BV, SVC, RA		Died 18 days
Muta (1977) [7]	F	37	No	At surgery	Papillary cell carcinoma	BV	Thrombectomy	Not reported
Thompson (1978) [8]	F	67	Yes	Venography	Follicular cell carcinoma	JV, BV, SVC, RA	Thrombectomy	Alive 24 months
Perez (1984) [9]	F	48	No	Venography, CT	Follicular cell carcinoma	JV, BV, SVC	Thrombectomy	Alive 4 months with metastases
Sirota (1989) [10]	F	61	Yes	At autopsy	Papillary cell carcinoma	AV	EBRT, ^131^I	Died 8 months
Niderle (1990) [11]	M	57	Yes	Venography, CT	Follicular cell carcinoma	JV, BV, SVC, RA	Thrombectomy	Died 13 months
Thomas (1991) [12 ]	M	61	Yes	CT	Thyroid cancer (unspecified)	JV		Sudden death
Lalak (1997) [13]	F	68	No	At surgery	Follicular cell carcinoma	JV	Thrombectomy segmental resection JV	Alive 9 months
Patel (1997) [2]	F	79	Yes	CT	Papillary cell carcinoma	JV, SVC, BV, PV	Thrombectomy resection JV	Died postoperatively Day 12
Onaran (1998) [14]	M	48	No	CT	Hurthle cell carcinoma	JV, SCV	Thrombectomy Segmental resection JV	Died 12 months
	F	48	No	Ultrasound	Papillary cell carcinoma	JV	Segmental resection JV	Alive 37 months
	F	68	No	At surgery	Hurthle cell carcinoma	JV	Segmental resection JV	Alive over 36 months
Bussani (1999) [15]	F	67	Yes	At autopsy	Follicular cell carcinoma	JV	EBRT	Died 4 months
Wiseman (2000) [16]	M	84	No	CT	Thyroid cancer (unspecified)	JV, BV, SVC, RA	^131^I	Died 12 months
Mishra (2001) [3]	F	30	No	At surgery	Poorly differentiated papillary thyroid carcinoma	JV	Excision JV	Unknown
	F	32	No	Venography	Papillary carcinoma	BV, JV	Resection JV, shaved off BV^131^I	Alive 4 yrs 10 month
	F	36	No	At surgery	Poorly differentiated papillary carcinoma	JV	Excision JV Thrombectomy ^131^I	Alive 2 yrs 6 months
	F	36	No	CT	Poorly differentiated thyroid carcinoma	JV	Radical neck dissection	Died 4 days post-operatively
	M	60	Yes	CT	Undifferentiated papillary thyroid carcinoma	JV	Excision JV	Died 1 day post-operatively
Koike (2002) [17]	F	26	No	At surgery	Papillary cell carcinoma	BV, SVC	Thrombectomy	Alive 8 months
Sugimoto (2006) [18]	M	61	Yes	CT, MRI, Venography	Poorly differentiated papillary cell carcinoma	BV, SVC, RA	Excision BV, SVC Thrombectomy Vein graft	Died 12 days post-operatively of renal failure

## Materials and methods

The Royal Marsden Hospital serves as a tertiary referral unit for patients with thyroid disease and maintains a tumour registry of patients with thyroid cancer based on a confirmed histological report of thyroid malignancy. All clinical information at the time of presentation and at follow-up is entered at the time of consultation. We searched for patients with clinical, radiological and pathological evidence of occlusion of the great veins in the neck. Patients had to have a minimum follow-up of 2 years after initial treatment so as to assess the course of their disease following treatment. Patient records were reviewed with respect to clinical presentation, pathological features, treatment, recurrence and survival.

A Medline literature search was performed using the MeSH terms "superior vena cava obstruction" or "great vein infiltration" or "venous occlusion" and "thyroid cancer." We searched from 1980 since before that time articles were not consistently linked to MeSH terminology. We have included reports dating before 1980 if these were detailed in the articles uncovered in the search.

## Case presentations

### Case 1

**Figure 1:**
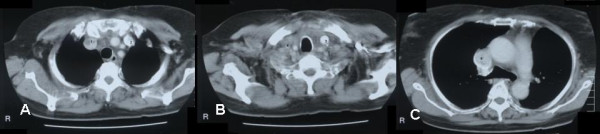
**(Case 1).1a **CT thorax showing filling defect in the right brachiocephalic vein (1) due to thrombus, while the left brachiocephalic vein (2) is patent and shows intense contrast enhancement. **1b **CT neck showing patent left internal jugular vein with intense contrast enhancement due to regurgitation (3). Right internal jugular vein is not seen due to thrombus (4). **1c **CT thorax showing blocked superior vena cava (5) with thrombus and a rim of contrast enhancement indicating partial block.

**Figure 2:**
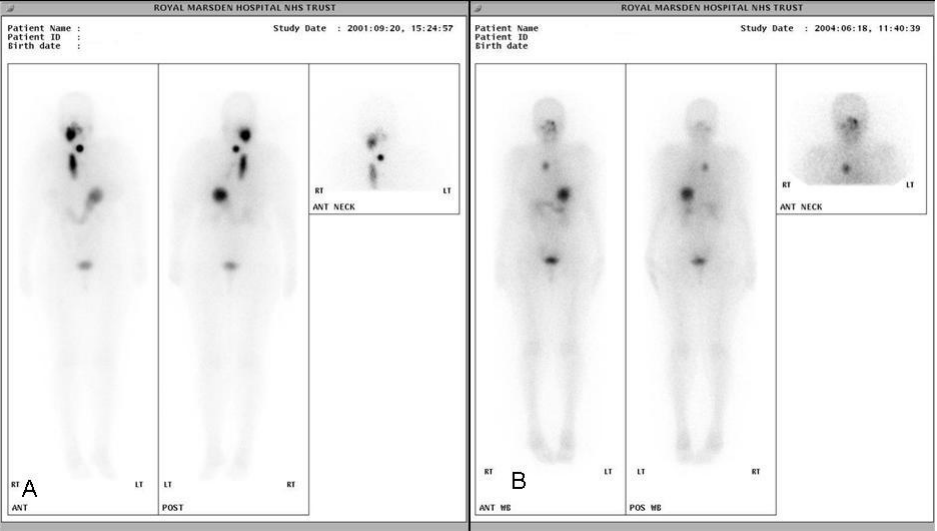
**(Case 1).2a**. Post radioiodine ablation scan showing abnormal ^131^I accumulation in the right upper neck and thyroid bed. There is a linear abnormality to the right of the midline of the upper chest extending inferiorly suggestive of residual tumour in the SVC. **2b**. Post radioiodine therapy scan showing a focus of intense ^131^I accumulation in the right upper mediastinum suggesting tumour at the root of the SVC. Marked improvement compared with initial scan (2a).

An 81 year old lady was referred for a painless mass arising in the right side of her neck of 4 month's duration. Cytology suggested follicular carcinoma. A staging computed tomography (CT) scan of the thorax performed pre-operatively showed a large smooth defect in the right brachiocephalic vein (Fig 1a). The right internal jugular vein (IJV) was completely blocked (Fig 1b) whilst thrombus extended and partially occluded the superior vena cava (SVC) (Fig 1c). At surgery there was evidence of tumour infiltration into the strap muscles extending up to the right submandibular gland and right IJV which was completely occluded. Total thyroidectomy and resection of the IJV were performed. Following surgery, she developed oedema of the face, neck, arms and bilateral breast engorgement. She was fully anticoagulated because a venous thrombus occluding the SVC could not be excluded. Histopathology confirmed that the IJV was infiltrated by multicentric follicular carcinoma. The cut end of the vein contained tumour. She was treated with ablative radioiodine (3GBq) plus radical dose external beam radiotherapy (EBRT) to the neck and superior mediastinum (total dose: 60 Gy). A post-ablation scan revealed streaky uptake of ^131^I within the right brachiocephalic vein extending to the superior vena cava (SVC) consistent with tumour thrombus (Figure 2a). Over the following 4 years, she received a total dose of 30GBq and repeat ^131^I scanning showed reduced uptake in the SVC (Figure 2b). Her symptoms had largely resolved.

Sixty four months after diagnosis she presented with diplopia and non iodine-avid skull metastases. She received palliative external beam radiotherapy (35Gy in 15 fractions). Her diplopia disappeared but she finally succumbed to progressive metastatic disease 2 months later.

### Case 2

**Figure 3:**
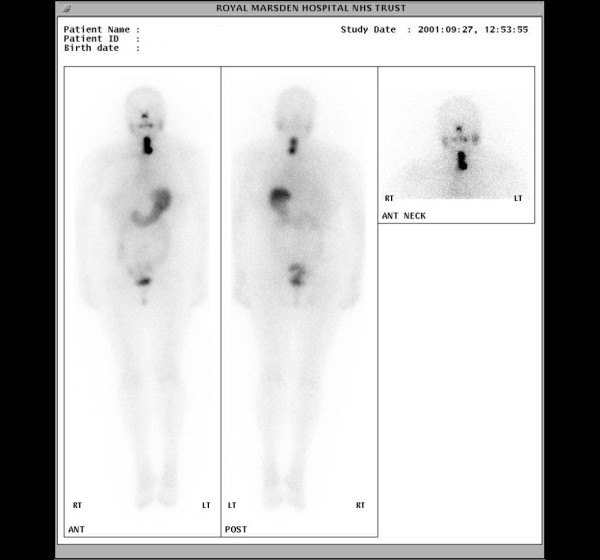
**(Case 2).** Radioiodine ablation scan showing a moderately sized area of accumulation to the right of midline of the lower neck corresponding to the internal jugular vein.

A lady aged 59 presented with a 9 cm right sided painless neck mass and right recurrent laryngeal palsy. A magnetic resonance (MR) scan of the neck performed by the referring physician showed a mass with high signal intensity arising from the right lobe of the thyroid, displacing the trachea and encasing the right IJV. Right cervical lymph nodes were enlarged from levels 2–4. At operation a highly vascular tumour was present extending down into the superior mediastinum, compressing and displacing the IJV and right brachiocephalic vein. Total thyroidectomy and neck dissection were performed with sacrifice of the IJV because of extensive encasement by tumour. Pathology revealed a widely infiltrating follicular carcinoma of the thyroid with tumour at the resected margins. Extensive lymphovascular and perineural invasion was noted, with tumour extending into the resected IJV. She received ablative ^131^I (3GBq) followed by a therapeutic dose of 5.8 GBq (Fig 3). Adjuvant EBRT was administered to the both sides of the neck, encompassing the extent of the original tumour to a total dose of 66 Gy in 33 daily fractions.

Twelve months following presentation, she developed a diffuse large B-cell lymphoma and was treated with CHOP chemotherapy. The patient died of cardiac failure but free of thyroid cancer (undetectable serum thyroglobulin) and free of lymphoma 23 months after presentation.

### Case 3

**Figure 4:**
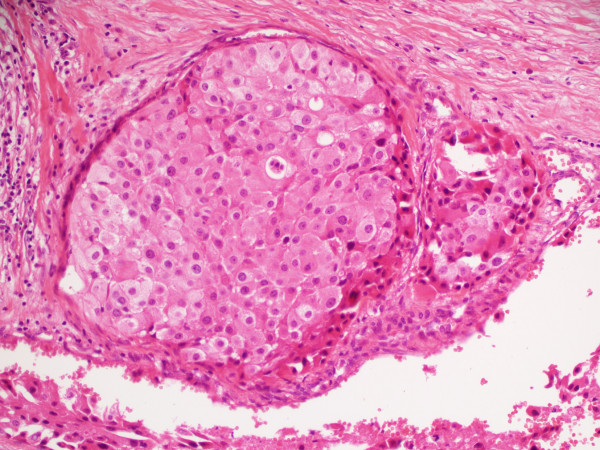
**(Case 3).** Hürthle cell carcinoma expanding right IJV lumen, with adjacent smaller tumour mass. Note cells with uniform round nuclei and abundant granular cytoplasm (haematoxylin and eosin × 200).

This 61 year old lady presented with a right sided painless hard thyroid swelling. A right thyroid lobectomy with right levels 3, 4 and 6 lymph node dissection was performed followed by completion thyroidectomy. At operation tumour was seen to be surrounding and invading the right IJV. Pathology revealed a 4 cm Hürthle cell carcinoma invading the right IJV with widespread infiltration of venules and veins (Fig 4). One of 8 lymph nodes was positive for tumour. A post-operative isotope scan showed no significant ^131^I uptake in the thyroid bed or elsewhere so she was not offered ablative ^131^I. She received radical dose EBRT to the neck and upper mediastinum. Her disease progressed and she developed brain metastases for which she received palliative radiotherapy with good results. She died of tumour 28 months after presentation.

### Case 4

This 43 year old lady presented with a firm left sided swelling in the neck. Staging CT and MRI of the neck were performed to assess operability. The scans showed a mass arising in the left lobe of the thyroid extending to the superior mediastinum. Multiple lymph nodes were visualized in the left cervical chain encasing the carotid sheath. At operation the left lobe of the thyroid was enlarged and adherent to the strap muscles, oesophagus and trachea, with retrosternal extension. A tubular mass of tumour was found to be invading the IJV and most of the associated venous complex in the upper neck extending up the common facial vein at the margin of the mandible. Tumour extended into the lumen of the deep lingual vein and other veins associated with the superior thyroid pedicle.

**Figure 5:**
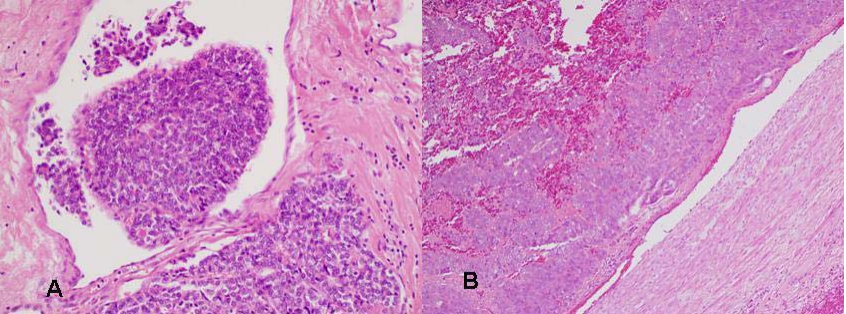
**(Case 4).5a) **Large, partially endothelialised direct extension of follicular carcinoma, attached to vessel wall (haematoxylin and eosin × 200). **5b) **Follicular carcinoma abutting wall of internal jugular vein (haematoxylin and eosin × 40).

**Figure 6:**
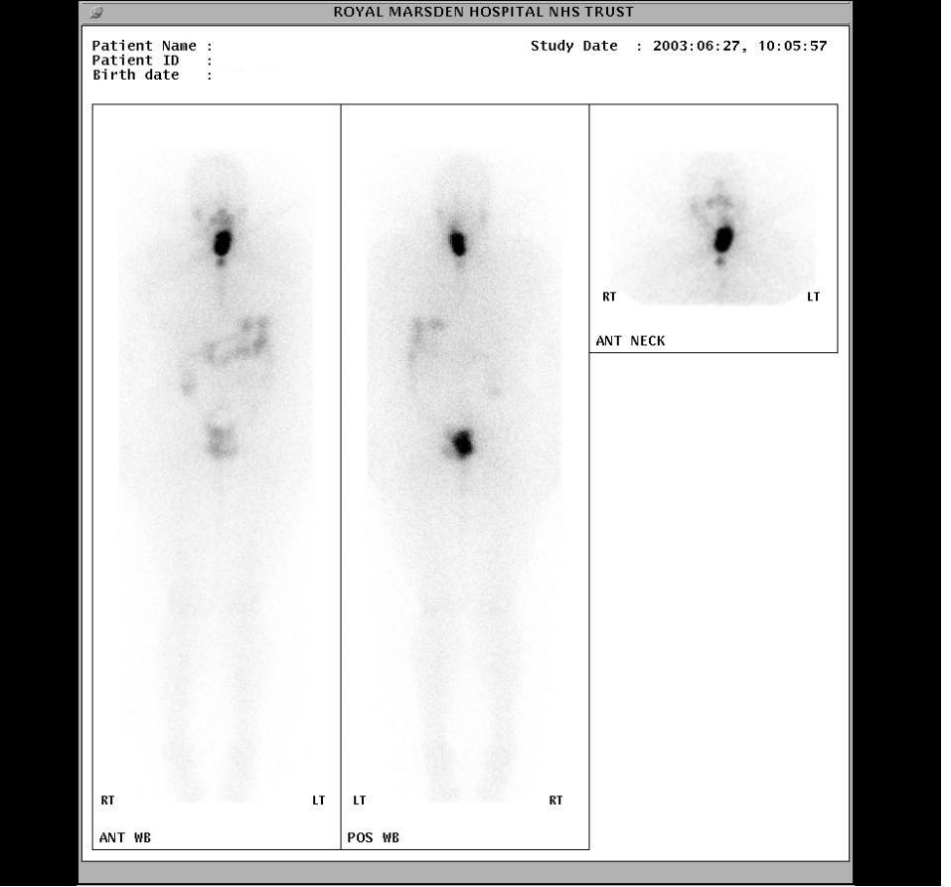
**(Case 4).** Ablation radioiodine scan showing a large area of accumulation in the midline of the neck with a further small low-grade focus inferior in the midline, suggestive of remnant thyroid or tumour tissue.

Total thyroidectomy with clearance of lymph nodes in levels 1,2, 3 and 4 was performed. The surgeon was able to dissect tumour free of the trachea and oesophagus but unable to conserve the left sternomastoid, left IJV, deep lingual and common facial veins, all of which were sacrificed. Pathology revealed a poorly differentiated follicular thyroid carcinoma. A mass of tumour was demonstrated in the resected IJV (Fig 5). Post-operative ^131^I scanning showed intense ^131^I accumulation in the midline of the neck (Fig 6).

Following surgery radical dose EBRT consisting of 46 Gy given in 23 fractions over four and a half weeks was administered to both sides of the neck up to the level of the mastoid processes, followed by 20Gy to the left side of the neck. In addition she received an ablative ^131^I dose of 5.5GBq followed by a further 5.6GBq therapeutic dose. Thirty three months after presentation, she developed cavernous sinus thrombosis with a tumour deposit in this area on MRI plus multiple lung and bone metastases. She received EBRT to the base of the skull with good symptomatic relief and remains asymptomatic but with disease 53 months after initial presentation.

### Case 5

This 70 year old gentleman presented with hoarseness followed by dyspnoea and progressive engorgement of neck veins. A nodular goiter was present on examination. CT scan revealed a bulky tumour in the thyroid bed extending into the pre-tracheal space and down the superior mediastinum to the level of the tracheal bifurcation. There was significant compression of the SVC from bulky right hilar lymphadenopathy. Lymphadenopathy was present from the angle of the mandible to the right supraclavicular fossa.

**Figure 7:**
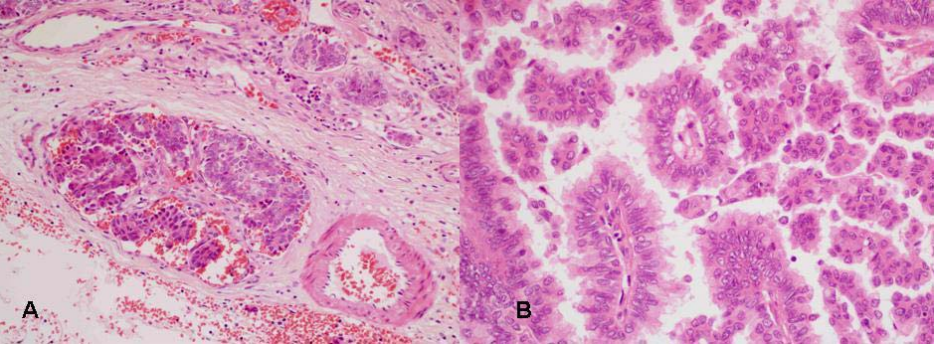
**(Case 5).7a) **Papillary carcinoma: papillary clusters of cells replacing large vessel with similar invasion of smaller vessels, top right (haematoxylin and eosin × 100). **7b) **Papillary carcinoma higher magnification: papillae with fibrovascular cores, lined by crowded cells with nuclear clearing and occasional grooving (haematoxylin and eosin × 400).

Pathology revealed a high grade papillary thyroid carcinoma with columnar cell architecture (Fig 7). He underwent total thyroidectomy and neck dissection followed by ^131^I ablation.

He received 5.5GBq ^131^I ablation followed by a 9.2GBq therapeutic dose. His symptoms improved initially but within 9 months hoarseness and engorgement of veins recurred. CT showed recurrent tumour in the neck and serum thyroglobulin rose from undetectable (post-operatively) to 4551 μg/l.

Hyper-fractionated accelerated radiotherapy to the neck and mediastinum delivered with a total dose of 50 Gy given in two phases: Phase 1 consisted of 40 Gy in 24 fractions to the neck and mediastinum twice daily; phase 2 comprised 10Gy in 6 fractions twice daily avoiding the spinal cord. There was some improvement in his symptoms and he died with locally controlled disease 26 months following presentation.

## Discussion

Obstruction of venous return in the mediastinum and neck is caused by a malignant process in up to 90% of cases, most commonly lung cancer [4]. However, it is rare for thyroid cancer to result in occlusion of the great veins either by extrinsic compression or tumour invasion of the venous wall and thrombosis. To date only 24 cases of thyroid cancer and invasion of mediastinal veins have been reported as shown in Table 1[2,3,5-18]. Of these, fifteen were treated aggressively with resection of the primary cancer and tumour thrombectomy. Five of these patients died within 12 days of surgery from post-operative complications; eight were alive at follow-up 4–58 (median 27) months, and outcome in two patients is not documented. The eight patients not aggressively treated had a median survival of 39 days following presentation.

In our series of 5 patients, all underwent total thyroidectomy and neck dissection. Where tumour was encasing or invading the jugular veins in the neck, it was resected. Ablative and therapeutic doses of radio iodine were given to all patients except in case 3 who had Hürthle cell carcinoma and no significant ^131^I uptake. Extensive tumour was present threatening major structures in the neck. It was decided that a complete response to ablative radioiodine could not be assumed and that waiting six months without further treatment before being able to give a therapeutic dose of ^131^I might prove hazardous. All patients were therefore treated with EBRT. We found a median survival of 28 months (range 23–66) and median disease free survival of 24 months (range 9–33) as shown in Table 2.

**Table 2:** Clinicopathological characteristics and prognosis

**Case**	**Sex**	**Age**	**Pathology**	**Vein involvement**	**Treatment**	**Survival (Months)**	**Disease Free survival (months)**
1	F	81	Follicular carcinoma	IJV, SVC, BCV	Surgery + EBRT + anticoagulation + ^131 ^I	66	30
2	F	59	Follicular Carcinoma	I JV	Surgery + EBRT + ^131 ^I	23	20
3	F	61	Hurthle cell Carcinoma	IJV	Surgery + EBRT	28	24
4	F	43	Poorly diff papillary carcinoma	IJV, Facial, Lingual	Surgery + ^131 ^I + EBRT	53*	33
5	M	70	Papillary carcinoma	SVC	Surgery + ^131 ^I + EBRT	26	10

Our patients had varying degrees of venous obstruction ranging from radiological signs only (cases 1, 2 and 4) or an incidental finding at surgery (case 3), to a florid SVC occlusion syndrome (case 5). This reflects the ability of venous collateral pathways to divert blood away from an obstruction.

The presence of dilated veins on the neck and torso is suggestive and was documented in 12 of the 24 reported cases (Table 1). Patients may complain of breathlessness, cough, headache and syncope. Thrombus may obstruct flow in associated veins such as external jugular or brachiocephalic veins giving rise to distinct clinical features [17]. Extension into the atria may cause sudden death in these patients [18].

CT scanning and MRI may differentiate external compression from intraluminal tumour. Intrathoracic extension of tumour should raise suspicion of involvement of the great vessels and should alert the surgeon to the possibility that a sternotomy or cardiopulmonary bypass may be required. In case 1, tumour thrombus was suggested by a smooth defect in the brachiocephalic vein extending into the SVC. A surrounding hypodense rim of blood clot may be also be demonstrated by CT. External compression was also correctly identified by CT in case 5. Encasement but not vascular invasion was seen on MRI in case 2. However in case 4, neither CT nor MRI demonstrated occlusion of the left IJV, deep lingual and common facial veins.

Colour Doppler ultrasound and venography may be helpful especially for excluding thrombus in the upper extremities but the SVC may be obscured by osseous structures or lung parenchyma [19]. CT venography has the advantage over digital subtraction venography in its ability to evaluate the proximal extent of obstruction or thrombosis [20]. Gallium-67 scintigraphy has been used successfully in diagnosing tumour thrombus in a patient with anaplastic thyroid cancer [21].

Complete resection is recommended where possible to reduce tumour burden. The presence of massive intravascular invasion should not be a contraindication for resection to palliate impending SVC obstruction [3]. Without surgery the prognosis is bleak and death follows from tumour embolism or obstruction of the right atrium [18]. During segmental vein resection, the involved vein is ligated before handling to prevent tumour embolisation [9]. Surgery should be complemented with radioiodine in iodine-avid tumours as this may reduce the risk of recurrence.

The value of EBRT in the management of thyroid cancer remains controversial because published data are conflicting and there are no prospective randomised controlled trials. There is good evidence that EBRT improves local control in patients with gross macroscopic residual disease following surgery [22]. In patients with residual microscopic disease postoperatively, a beneficial effect of EBRT was reported in patients with papillary thyroid cancer [23]. We recommend EBRT for all patients with known microscopic disease following surgery if older than 45 years or if tumour is poorly differentiated, and for known macroscopic disease [24]. It is also recommended for advanced and recurrent Hurthle cell carcinoma as this tumour takes up iodine infrequently [25]. The maximum dose of EBRT with acceptable toxicity was 60 Gy over 6 weeks in this series similar to that previously reported [26]. Venous obstruction by thyroid cancer occasionally responds dramatically to EBRT [27].

The circulation is well compensated by collaterals in patients with long standing venous obstruction and surgery is generally well tolerated. Stenting as a palliative therapy can be considered if surgery is not feasible [28]. Patients with rapidly progressing compression symptoms should be offered symptomatic treatment in the form of bed rest, oxygen and corticosteroids.

## Conclusion

Our small number of patients makes it impossible to propose a treatment based on evidence. A prospective randomised trial comparing different treatment modalities would provide reliable evidence but this is not feasible with such a rare condition. Despite this difficulty, multimodality therapy which includes surgery, radioiodine and external beam radiotherapy appears to offer the best chance of prolonging survival.

## Abbreviations

SVC – Superior vena cava IJV – Internal jugular vein EBRT – External beam radiotherapy ^131^I – Radioiodine therapy

## Competing interests

The author(s) declare that they have no competing interests.

## Authors' contributions

HSL: Final draft and literature review, PD: Clinical information, initial draft, NK: Discussion and editing, HM: Clinical information, CT images and interpretation, TK: Pathological images and reports, WK: Scintigram images and interpretation, HCL: Original concept, final editing. All authors read and approved the final manuscript.
